# Open necrosectomy combined with continuous positive drainage and prophylactic diverting loop ileostomy for late infected pancreatic necrosis: a retrospective cohort study

**DOI:** 10.1186/s12876-020-01343-7

**Published:** 2020-07-08

**Authors:** Dong-Guang Niu, Wei-Qin Li, Qian Huang, Fan Yang, Wei-Liang Tian, Chen Li, Lian-An Ding, Hong-Chun Fang, Yun-Zhao Zhao

**Affiliations:** 1 Department of General Surgery, Jinling clinical college of Nanjing Medical University, Nanjing, 210002 Jiangsu China; 2 Gastrointestinal Surgery Department, Affiliated Hospital of Qingdao University, Qingdao, 266000 Shandong China; 3 Oncology Department, Xintai people’s Hospital, Tai’an, 271200 Shandong China; 4 Department of General Surgery, Jinling clinical college of Nanjing Medical University, 305 East Zhongshan Road, Nanjing, 210000 Jiangsu China

**Keywords:** Infected pancreatic necrosis, Open necrosectomy, Prophylactic diverting loop ileostomy, Continuous positive drainage

## Abstract

**Background:**

To evaluate an innovative open necrosectomy strategy with continuous positive drainage and prophylactic diverting loop ileostomy for the management of late infected pancreatic necrosis (LIPN).

**Methods:**

Consecutive patients were divided into open necrosectomy (ON) group (*n* = 23), open necrosectomy with colonic segment resection (ON+CSR) group (*n* = 8) and open necrosectomy with prophylactic diverting loop ileostomy (ON+PDLI) group (*n* = 11). Continuous positive drainage (CPD) via double-lumen irrigation–suction tube (DLIST) was performed in ON+PDLI group. The primary endpoints were duration of organ failure after surgery, postoperative complication, the rate of re-surgery and mortality. The secondary endpoints were duration of hospitalization, cost, time interval between open surgery and total enteral nutrition (TEN).

**Results:**

The recovery time of organ function in ON+PDLI group was shorter than that in other two groups. Colonic complications occurred in 13 patients (56.5%) in the ON group and 3 patients (27.3%) in the ON+PDLI group (*p* = 0.11). The length of stay in the ON+PDLI group was shorter than the ON group (*p* = 0.001). The hospitalization cost in the ON+PDLI group was less than the ON group (*p* = 0.0052).

**Conclusion:**

ON+PDLI can avoid the intestinal dysfunction, re-ileostomy, the resection of innocent colon and reduce the intraoperative trauma. Despite being of colonic complications before or during operation, CPD + PDLI may show superior effectiveness, safety, and convenience in LIPN.

## Background

Severe acute pancreatitis (SAP) is a serious disease involving multiple disciplines and systems. A certain proportion of patients with SAP would develop infected pancreatic necrosis (IPN) in the later course of the disease [1]. IPN is a severe complication of SAP with mortality at about 30% (12–39%) [2–5]. As the treatment protocols sufficiently addressed the under lining pathophysiological mechanisms of the disease, the step-up approach with minimally invasive techniques is emerging as the main stream in the appropriate IPN patient [6, 7]. However, less than 20% patients with LIPN who have failed in minimally invasive surgery still need to be treated with ON ultimately [8]. The operations used for LIPN are aimed at removing necrosis or devitalized tissue, draining pus, providing a safer avenue for egress of pancreatic secretions and the leakage of gastrointestinal tract and managing the colonic complications. Recurrent post necrosectomy local sepsis, due to inadequate drainage, continues to pose a major drawback [9, 10]. In our central, continuous positive drainage (CPD) via double-lumen irrigation–suction tube (DLIST) is one of the key techniques by insert the DLIST into abscess, focus of necrotic and abdominal cavity during the procedure of ON for postoperative CPD, which could ensure the adequate drainage. For colon complications, resection with proximal ostomy and diverting loop ileostomy (DLI) constitutes the treatment for suspected imminent or overt ischemia/perforation in majority of cases [11, 12]. However, just evaluating the outer aspect of the colon, identification of colon involvement may be difficult due to nonspecific symptoms or be masked by the sepsis. On one hand, 45.4% patients were detected with GI fistula after performing open necrosectomy. DLI, ileostomy or colostomy was performed for 65.3% colonic fistulas [13, 14]. On the other hand, with a low threshold for colonic resection due to unreliable detection of ischemia or imminent perforation by outside inspection during surgery for IPN, histologically examined specimens showed that colonic resection was unnecessary in 20–50% [12, 15, 16]. Postoperative mortality was as high as 50% [17]. However, up to now, comparing the role of DLI and the aggressive form of treatment such as subtotal/segment colectomy in terms of clinic outcome and prognosis, there is no data to suggest that which one is more advantageous in treating IPN patients with the suspected imminent or overt ischemia/perforation before or during operation. Also, there is no report about the merits of PDLI in ON for IPN without colonic complications before or during operation.

Therefore, in current study, we aimed to evaluate the effectiveness, safety, and convenience of CPD + PDLI in LIPN compared to the other two open necrosectomy approaches, no matter with or without colonic complications before or during operation.

## Methods

### Patients

From January 2012 to February 2017, all the consecutive patients admitted to our center with a diagnosis of IPN were registered in an internal database and screened for potential enrollment. Patients who were performed open pancreatic necrosectomy and diagnosed with IPN during the study period were collected. The data were assembled and analyzed retrospectively. Informed consent was exempted because this retrospective study was harmless to the patients and contained no personal data. The study was approved by the Institutional Review Board of Jinling hospital.

The inclusion criteria for the study were: (1) patients diagnosis with IPN according to the presence of gas bubbles within pancreatic necrosis on contrast-enhanced CT scan or a positive bacterial culture obtained by fine-needle aspiration, first drainage and/or operation [18]; (2) patients performed with open pancreatic necrosectomy and diagnosed with IPN.

The exclusion criteria were: (1) pregnant patients; (2) patients received chemotherapy for malignancy or auto-immune diseases; (3) patients received abdominal surgery before IPN and was present due to abdominal compartment syndrome (ACS), perforation of a visceral organ, bleeding during the current episode of AP; (4) patients received ON for IPN before admitting to our institute during the current episode of AP; (5) IPN was caused due to trauma (6) the treatment strategy was not completed for nonmedical reasons.

Initial medical treatment and minimally invasive step-up approach were carried out for every patient before and after IPN confirmation according to the international recommendations [6, 19].

### Endpoints

For each participant, the following variables were collected, including age, sex, etiology, and body mass index (BMI), time interval between AP onset to operation. Baseline characteristics such as acute physiology and chronic health evaluation (APACHE) II score, sequential organ failure assessment (SOFA) score, laboratory data including C-reactive protein (CRP), procalcitonin (PCT), Interleukin-6 (IL-6) and white blood cells (WBC) were also collected and assessed within 24 h before surgery, at the third and seventh day after surgery.

Our primary endpoints were duration of organ failure after surgery, postoperative complication, the rate of re-surgery and mortality. Secondary endpoints were duration of hospitalization, cost, time interval between open surgery to total enteral nutrition (TEN). The main postoperative complications included colonic complication, hemorrhage, pancreatic fistula. The colonic complications included colonic suspected imminent or overt ischemia/perforation, stenosis, hemorrhage, colonic fistula, pseudo-obstruction.

Organ functions were evaluated in cardiovascular, renal and respiratory systems. The criteria for cardiovascular, renal and respiratory failure were defined based on international consensus [18, 20], cardiovascular (systolic blood pressure<90 mmHg despite adequate fluid resuscitation or need for inotropic agent), renal [serum creatinine ≥171 μmol/L (2.0 mg/dL) after rehydration and respiratory [PaO2/FiO2 ≤ 300 mmHg (40 kPa)].

Gastrointestinal fistula was defined as the discharge of the gastric contents, bowel from drain or surgical wound. Intra-abdominal bleeding was defined as peritoneal/retroperitoneal bleeding that required surgical, radiologic or endoscopic treatment. Gastrointestinal bleeding was defined as that the blood loss from the mouth to the rectum was more than 500 ml/24 h.

### Minimally invasive step-up approach

Percutaneous or endoscopic transgastric drainage was firstly performed. The preferred route was through the left retroperitoneum, thereby facilitating minimally invasive retroperitoneal necrosectomy at a later stage. If there was no clinical improvement after 72 h and if the position of the drain was inadequate or other fluid collections could be drained, a second drainage procedure would be performed. If this was not possible, or if there was no clinical improvement after an additional 72 h, then, video-assisted retroperitoneal débridement with postoperative lavage, was performed.

### Treatment

**Fig. 1:**
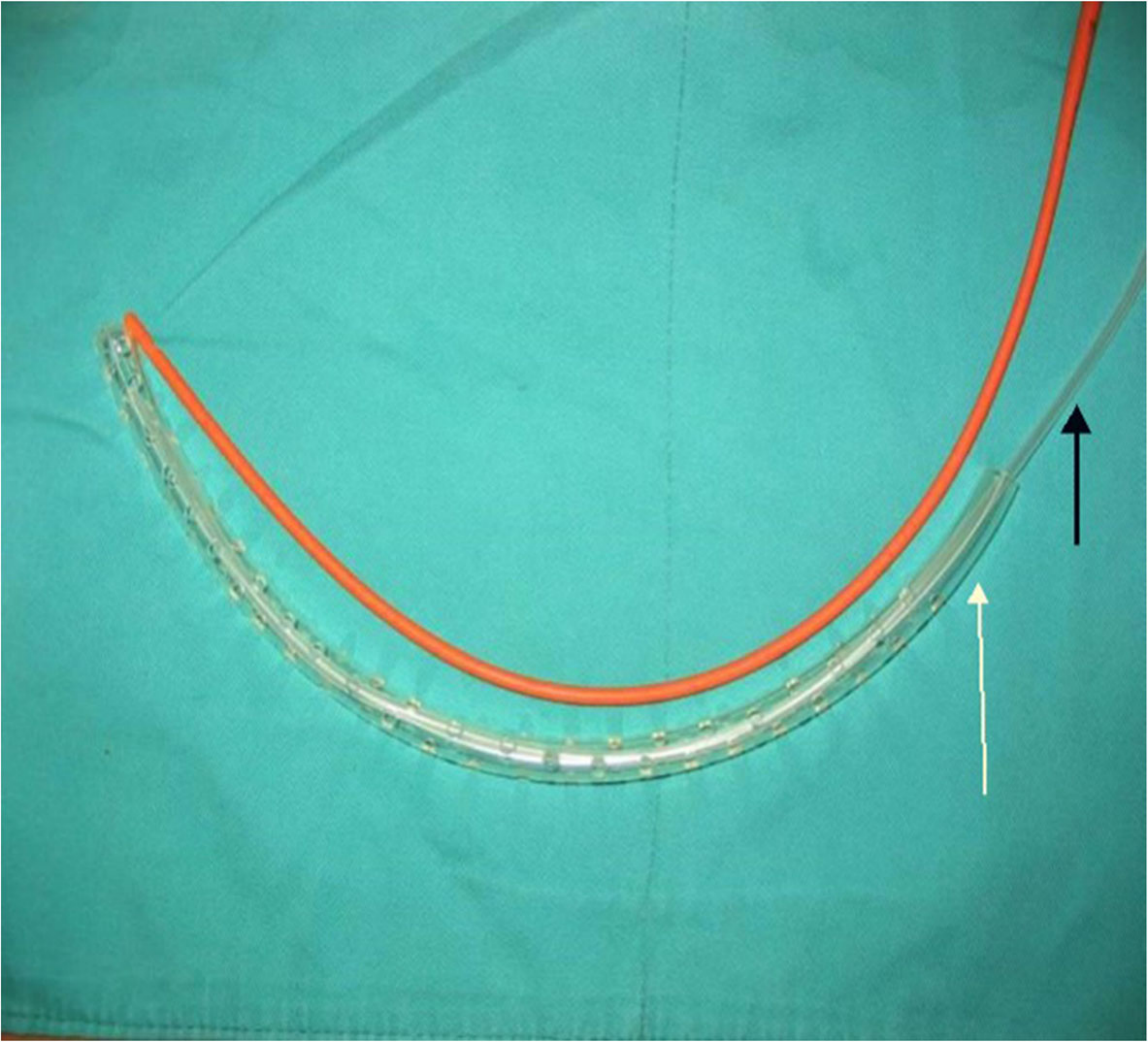
Double-lumen irrigation–suction tube (DLIST) consists of a red irrigation catheter, a suction catheter (identified by black arrow), and a supporting pipe (identified by white arrow). It works under continuous negative pressure of 150 to 200 millibars along with continuous saline irrigation at 300 mL/h

Three ON strategies were used in the management of LIPN. Among them, continuous positive drainage via a large-bore double-lumen irrigation-suction tube is one of the key techniques. The significance of positive drainage system has been confirmed by several studies [4, 13, 14]. The procedure of ON consist of a laparotomy through a bilateral subcostal incision, after blunt removal of pancreatic and peri-pancreatic necrosis, several large-bore double-lumen irrigation-suction tube inserted for postoperative CPD (Fig. 1).

Additionally, jejunostomy was performed for enteral feeding and the abdomen was closed. The first group patients without suspected imminent or overt ischemia/perforation before or during operation were performed ON alone. The second group patients associated with suspected imminent or overt ischemia/perforation before or during operation underwent colonic segmental resection (CSR) with proximal ostomy, as well as ON. The third group patients, in addition to ON, underwent PDLI, despite suspected imminent or overt ischemia/perforation before or during operation. According to the different surgical procedures, they were divided into three groups, ON, ON+CSR, and ON+PDLI.

### Statistical analysis

SPSS software (SPSS for Windows, version 23.0, SPSS, Chicago, IL) was applied for statistical analysis. Measurement data was presented as median and interquartile range (IQR). Kruskal-Wallis was performed to compare variance among three groups and Bonferroni correction was used to compare two groups. For categorical variables, chi-squared test was performed to compare the constituent ratio among the three groups. Fisher’s exact test was performed between two groups. *P* < 0.05 was considered as statistically significant.

## Results

### Patient characteristics

**Fig. 2:**
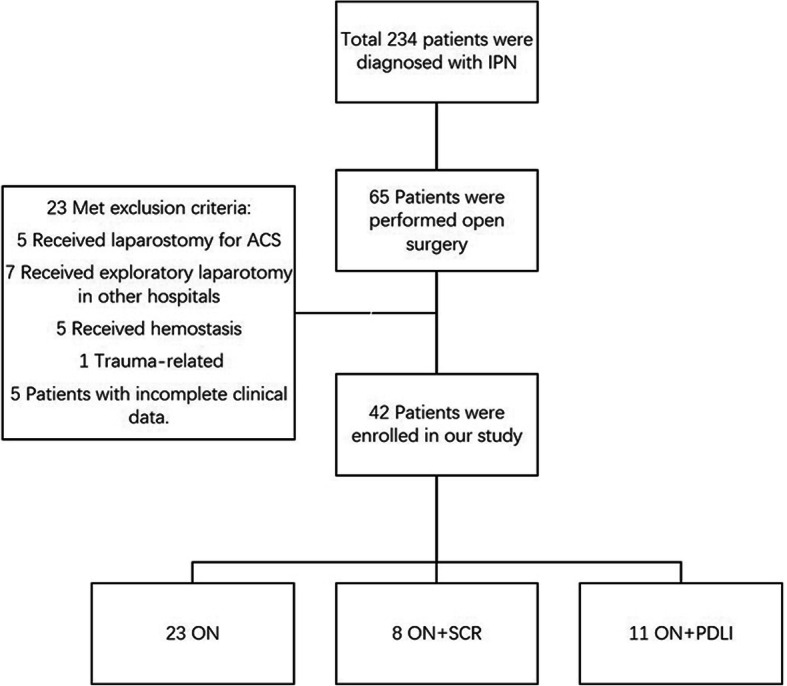
Screening and enrollment of the study patients

From January 2012 to December 2017, a total of 234 patients with IPN were admitted to our hospital, of which 65 patients were performed with open surgery. Forty-two patients were treated with ON for LIPN after failed minimally invasive approach were enrolled in the retrospective cohort study. The other 23 patients were excluded as the following reasons. Five patients underwent laparostomy for ACS, 5 patients underwent laparotomy for abdominal hemorrhage, 1 patient was trauma-related, 7 patients received exploratory laparostomy in other hospital and 5 patients with incomplete clinical data. (Fig. 2).

All the enrolled 42 patients were divided into three groups, 23 patients in ON group, 8 in ON+CSR group and 11 in ON+PDLI group, according to the different surgical procedures. Patients in the three treatment groups had similar demographic and clinical characteristics. The major disease causes of these patients included biliary disease, alcohol and hyperlipidemia. There were no significant differences of age, gender, BMI, smoking history, and history of alcohol intake in patients among three groups. (Table 1).

**Table 1:** Demographic characteristics

Characteristic	ON *N* = 23	ON+SCR *N* = 8	ON+PDLI *N* = 11	p
Age, median (IQR), year	54 (27–67)	41.5 (34.5–64.5)	47 (32–54)	0.638
Gender, male/female	15/8	4/4	5/6	0.587
BMI, median (IQR)	22.5 (20.7–25.4)	23.0 (20.5–24.95)	22.4 (20.6–24.3)	0.87
Pathogenesis (%)				
Biliary	11	4	5	1.00
Alcohol	6	2	3	1.00
Hypertriglyceridemia	4	1	3	0.759
Other	2	1	0	0.758
Nonmedical history				
Smoking	12	3	4	0.644
Drinking	8	2	4	1.00

Note: Kruskal-Wallis was performed to compare variance among three groups. For categorical variables, chi-squared test was performed to compare the constituent ratio among the three groups

### Comparison of inflammatory factors

**Fig. 3:**
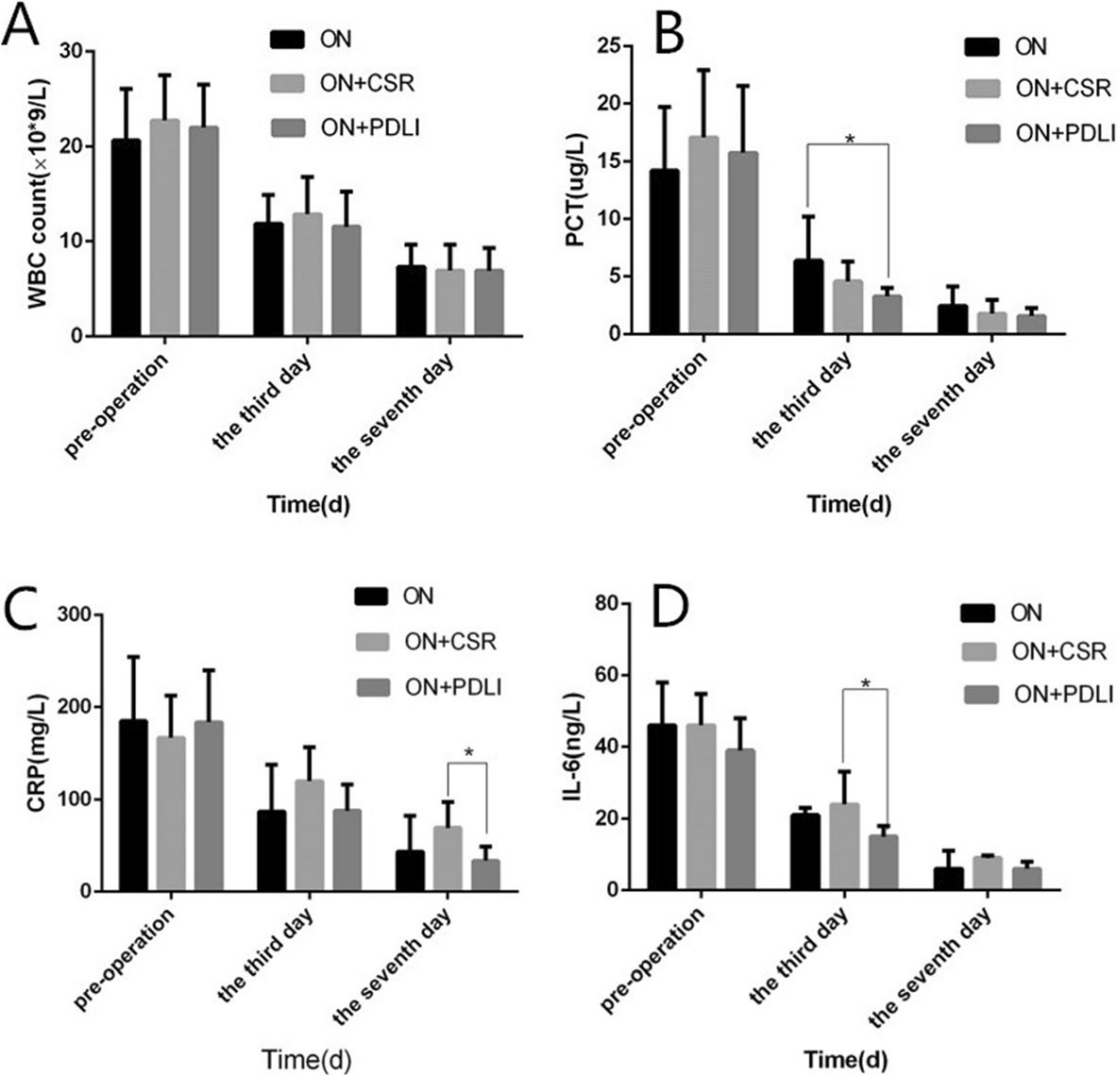
Comparison of laboratory indicators. **a**. WBC was decreased in all groups, but there was no significant difference in the three groups; **b.** The reduction of PCT in the ON+PDLI group was greater than that in the ON group at the 3rd day after operation; **c.** The CRP improvement in the ON+PDLI group was better than that in the ON+CSR group at the 7th day after operation; **d.** The reduction of IL-6 in the ON+PDLI group was better than the ON+CSR group at the 3rd day after operation

The inflammatory factors, including WBC, PCT, CRP and IL-6, in patients from all groups were lower than that before the treatment. As the course of treatment progressed, the level of the inflammatory factors decreased gradually. IL-6 at the 3rd day in the ON+PDLI group was remarkable lower than the ON+CSR group (*p* < 0.05). Meanwhile, at the 3rd day, PCT in the ON+PDLI group was also lower than the ON group (*p* < 0.05). At the 7th day, CRP in the ON+PDLI group was significantly lower than that in the ON group (*p* = 0.002). (Fig. 3).

### Comparison of treatment scores

**Fig. 4:**
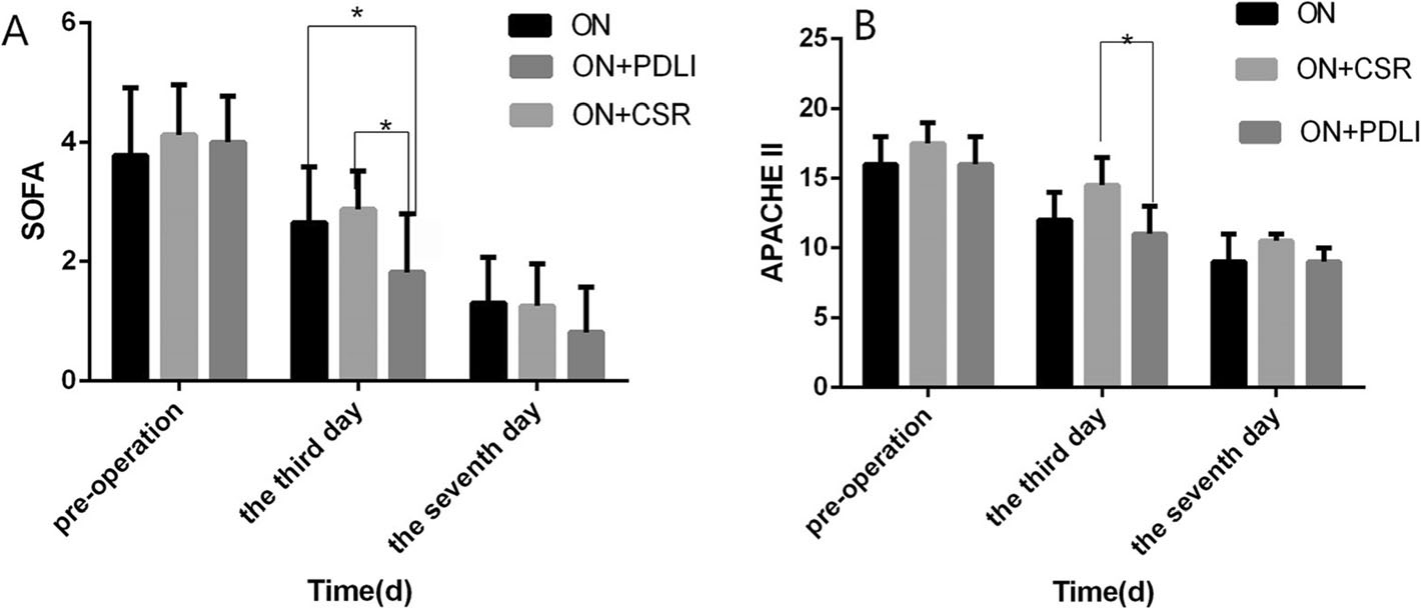
Comparison of treatment scores. **a.** SOFA score of the ON+PDLI group was better than that of the ON group and ON+CSR group at the 3rd day after operation; **b.** APACHE II score of the ON+PDLI group was better than that of the ON+CSR group at the 3rd day after operation

At the 3th day after surgery, the SOFA score in the ON+PDLI group was significantly lower than the ON group and ON+CSR group (*p* < 0.05). (Fig. 4a) APACHE II score showed a similar disease severity among patients within 24 h before open surgery in all groups [16.50 (IQR12–18) for ON group, 17.0 (IQR11–19) for ON+CSR group and 16.0(IQR9–18) for ON+PDLI group, *p* = 1.00)]. Our results showed systematic condition was improved in all groups after treatment. However, it was significant in the ON+PDLI group than in the ON+CSR at the 3th day after surgery (*p* < 0.05). (Fig. 4b).

### Primary endpoints

In ON group, 13 patients (56.5%) had colonic complication, including 2 patients with colonic fistula, 4 patients with colonic stenosis, 2 patients with colonic fistula, 2 patients with stenosis, 3 patients with pseudo-obstruction, 1 patients with duodenocolonic fistula and 1 patients with biliary-colon fistula. The complication rate decreased to 27.3% (3/11 patients) in ON+PDLI groups, consisting of colonic fistula in 1 case, colonic stenosis in 1 case and colonic fistula with stenosis in 1 case. The incidence rate of colonic complication in ON+PDLI group was lower than that in the ON, but there was no significant difference (*p* = 0.11). In the ON group, 7 cases underwent re-operative ileostomy or colostomy due to colonic complication. Comparing to the ON+PDLI group, the rate of re-surgery in ON group was significantly higher (*P* = 0.04). As for the other major complications including pancreatic fistula, hemorrhage, and death showed no significant differences in the three groups. In addition, in the ON+CSR group, 5/8 patients were colonic necrosis/perforation, 3/8 patients were pericolitis and fat necrosis, which were confirmed by histological examination.

**Fig. 5:**
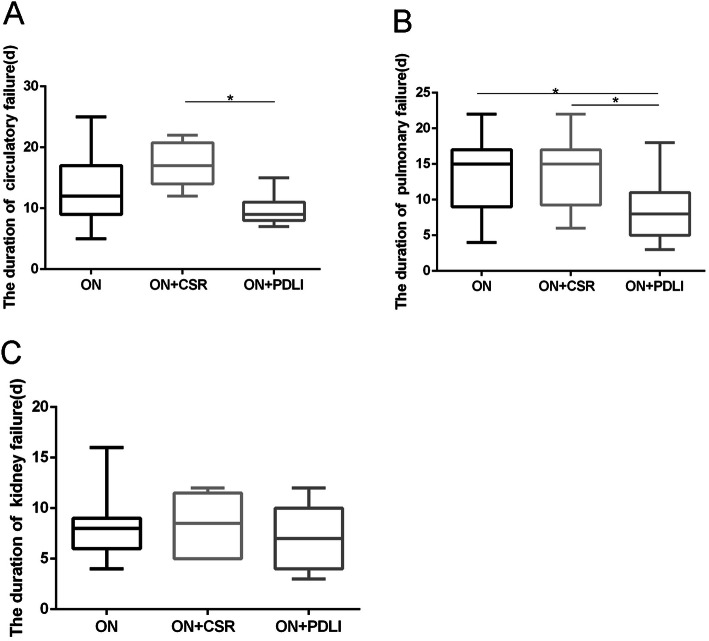
Comparison of during of the organ failure. **a** The duration of circulatory failure of the ON+PDLI group was shorter than that of the ON+CSR group; **b** The duration of pulmonary failure of the ON+PDLI group was shorter than that of the ON group and ON+CSR group after treatment

The duration of postoperative circulatory failure was 12 (9–17), 17 (14–20) and 9 (8–11) days in ON, ON+CSR and ON+PDLI groups respectively. A remarkable decrease of postoperative duration of circulatory failure existed in ON+PDLI group compared with ON+CSR group (*p* = 0.004). (Fig. 5a) Meanwhile, the duration of postoperative pulmonary failure in ON+PDLI group was also significantly shorter than that in ON group and in ON+CSR group (*p* = 0.012, *p* = 0.026, respectively). (Fig. 5b) There was no significant difference of duration of postoperative renal failure among the three groups (*p* = 0.438, Fig. 5c). (Table 2).

**Table 2:** Primary endpoints

	ON *N* = 23	ON+SC *N* = 8	ON+PDLI *N* = 11	p
The duration of postoperative circulatory failure, median (IQR), days	12 (9–17)	17 (14–20)	9 (8–11)	0.0068
The duration of postoperative pulmonary failure, median (IQR), days	15 (8–19)	16 (9–25)	8 (5–11)	0.026
The duration of postoperative kidney failure, median (IQR), days	8 (6–9)	8 (5–11.5)	7 (4–10)	0.438
new-onset colonic complication no. (%)	13 (56.5)		3 (27.3)	0.11
Colonic fistula	2		1	
Colonic stenosis	4		1	
Colonic fistula with stenosis	2		1	
pseudo-obstruction	3			
duodenocolonic fistula,	1			
biliary-colon fistula	1			
sequence ileostomy or colostomy no. (%)	7 (30.4)		0	0.04
Pancreatic fistula	11	3	4	0.75
hemorrhage	3	1	1	1.00
Intra-abdominal bleeding	2	1	1	
Gastrointestinal bleeding	1			
death no. (%)	3 (13.0)	2 (25)	1 (9.1)	1.00
Colonic necrosis/perforation confirmed by histological examination.		5		

Note: Kruskal-Wallis was performed to compare variance among three groups and Bonferroni correction was used to compare two groups. For categorical variables, chi-squared test was performed to compare the constituent ratio among the three groups. Fisher’s exact test was performed between two groups

### Secondary endpoints

The median time from AP onset to open surgery was 25 (19–35), 44 (34–54), and 22 (15–34) days in ON, ON+CSR and ON+PDLI groups respectively. The median days in ON+CSR group were significantly longer than that of in the ON (*p* = 0.0011) and ON+PDLI groups (*p* = 0.0069).

**Fig. 6:**
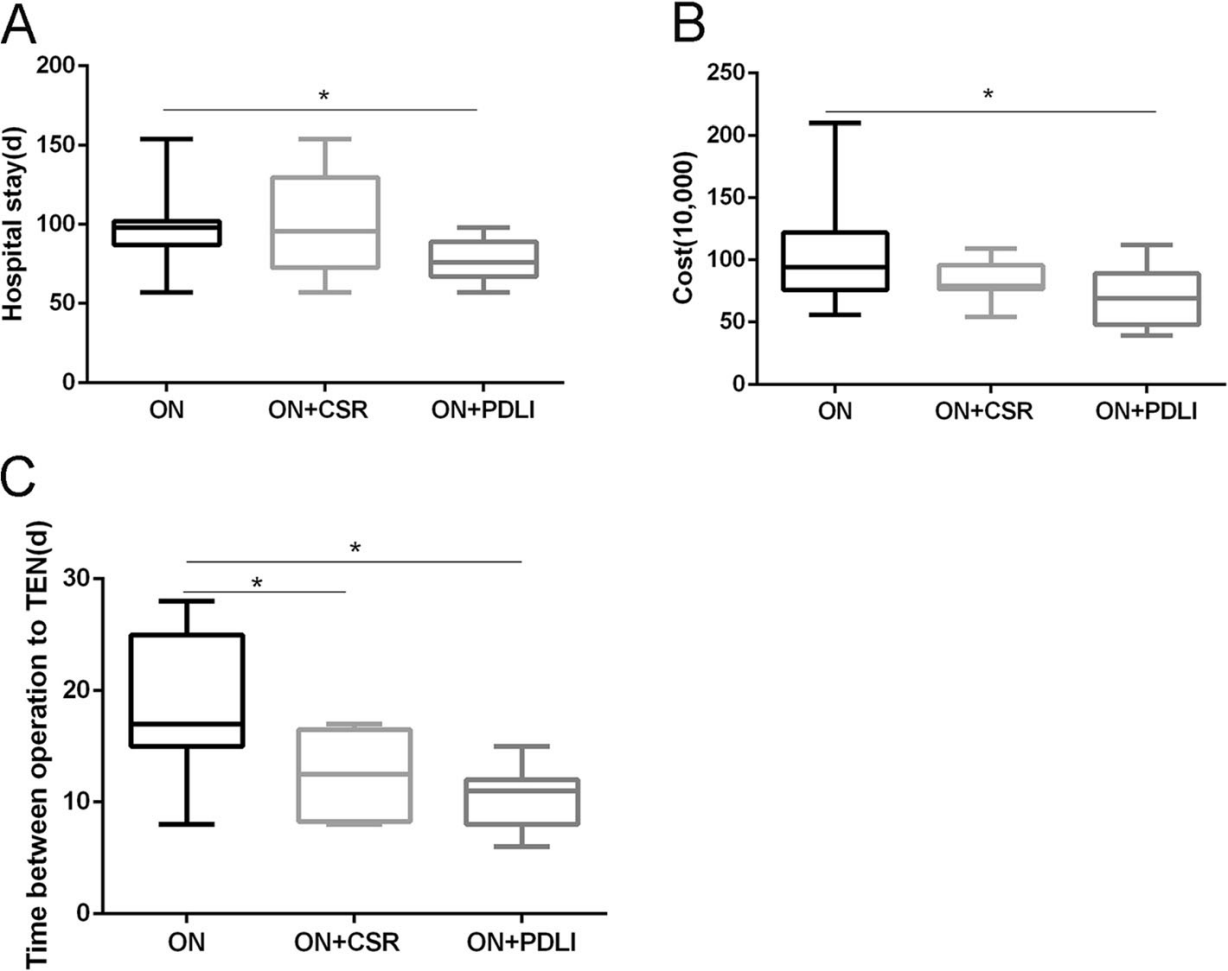
Comparison of clinical indicators. **a.** The length of stay in the ON+PDLI group was shorter than the ON group; **b.** The hospitalization cost in the ON+PDLI group was less than the ON group; **c.** The time interval between operation and TEN in the ON group was longer than the ON+CSR group and ON+PDLI group

The median hospital stays in ON group [98 (87–102) days] were similar to that of the ON+CSR group [95.5 (72.5–129.8) days], while the days in ON+PDLI group were significantly shorter than the ON groups (Fig. 6a). Higher hospitalization cost was observed in the ON group than that in the ON+CSR and ON+PDLI group, however, there was only significantly difference between the ON group and ON+DPLI group (*p* = 0.0052) (Fig. 6b). Additionally, it was notable that patients treated by ON approach had longer time interval between operation to TEN than the ON+CSR group and ON+PDLI group (*p* = 0.0085, p<0.0001 respectively) (Fig. 6c). (Table 3).

**Table 3:** Secondary endpoints

	ON *N* = 23	ON+SC *N* = 8	ON+PDLI *N* = 11	P
Time interval between AP onset to operation, median (IQR), days	25 (19–35)	44 (34–54)	22 (15–34)	0.0052
Days in hospital, median (IQR), days	98 (87–102)	95.5 (72.5–129.8)	76 (67–89)	0.0089
Cost, 10,000 median (IQR), CHY	94 (76–122)	79 (76–95)	69 (48–89)	0.0188
Time interval between operation to TEN, median (IQR), days	17 (15–25)	12.5 (8.5–16.5)	11 (8–12)	0.001

Note: Kruskal-Wallis was performed to compare variance among three groups

## Discussion

Severe acute pancreatitis (SAP) is a serious disease involving multiple disciplines and systems. IPN is a complication of SAP at its end stage, with a high mortality rate (14–69%) due to multiple organ failure, despite advances in critical care and surgical technique [21]. IPN is of great importance to surgeons, because medical management alone does not reduce mortality. With the development of minimally invasive treatment, step-up approaches combining new minimally invasive techniques seem to gradually replace the traditional ON and become the mainstream of IPN therapy, in which the major complications and mortality were lower compared with ON [6]. However, open surgery still plays an irreplaceable role in the treatment of SAP complications such as gastrointestinal fistula, hemorrhage, ACS, LIPN, 10–20% patients with IPN still underwent ON eventually [22]. The operations used for LIPN are aimed at removing dead or devitalized tissue, draining pus, providing a safer avenue for egress of pancreatic secretions and the leakage of gastrointestinal tract, and managing the colonic complications. For ON, inappropriate surgical procedures will aggravate the systemic pathophysiologic disturbances, sepsis shock, and MOF, which may cause catastrophic consequences or even death. The optimal ON techniques are of great significance in decreasing major post-operative complications, shortening hospital stay, and reducing the morbidity and mortality of patients [23]. Lavage system with large-bore drains constitute the most recommended option based on the reducing mortality, which is supported by the results of several randomized controlled trials [24, 25]. Our study confirmed that continuous positive drainage via a large-bore double-lumen irrigation-suction tube is the pivotal techniques in ensuring adequate drainage. None of the patients underwent reoperation because of local sepsis. More controversies came from the management of colon complications.

Several researches recommend a low threshold for colonic resection due to suspected imminent or overt ischemia/perforation by outside inspection during surgery. But 12.5–50% patients who were performed with aggressive surgical approach suffered innocent colonic resection without ischemia/perforation [12, 15, 16]. Just evaluating the outer aspect of the colon, identification of colon involvement may be difficult because of nonspecific symptoms or being masked by the sepsis. Borie D et al. [11] demonstrated that diverting loop ileostomy should also be performed in IPN when colonic viability was dubious. Up to now, comparing the role of DLI and the aggressive form of treatment such as subtotal/segment colectomy in terms of clinic outcome and prognosis, there is no data to suggest that which one is more advantageous in treating IPN patients with the suspected imminent or overt ischemia/perforation before or during operation. Additionally, many postoperative patients without suspected imminent or overt ischemia/perforation before or during surgery have got colonic complications such as stenosis, pseudo-obstruction, and even new-onset ischemia/perforation.

Sustained colonic complications may prolong parenteral nutritional support, which is attended by potential poor prognosis [26]. Many researches reported colonic fistulas occuring in 15–53% patients after necrosectomy and drainage [27–29]. In addition to pancreatic fistula, colonic fistulas were the second frequently postoperative complication of ON [30, 31]. Mohamed SR et al. advocate surgery for colonic fistulae, which have the worst outcome, possibly due to local sepsis [32]. Jiang et al. reported sequence ileostomy or colostomy was performed for 61.1% (44/72) colonic fistula [15]. As showed in our ON group, 17.4%(4/23) cases suffered post-operation colonic fistulae. Re-operation ileostomy or colostomy was performed 53.8%(7/13) for colonic complications. Untimely diagnosis of colon complications is a main cause of prolonged hospital stay, which increased costs and high mortality [33].

In the current study, WBC, CPR, PCT and IL-6 were chosen as indicators of the infection and inflammatory response [34–36]. The results showed that patients treated with ON+PDLI experienced a more rapid improvement in inflammation over time, especially on the third day after operation. Therefore, we consider that ON+PDLI can improve systemic infection, and reduce the inflammatory response promptly. According to the APACHE II score and the SOFA score, there was no significant difference of the severity of disease among patients in the three groups within 24 h before operation. Compared with ON, ON+PDLI did not increase the damage of patients. Consistent with inflammatory factors, the recovery time of organ function in ON+PDLI group was shorter than that in other two groups. The ON+PDLI was more aligned with the damage control principal [37]. The operation time, complexity and traumatic stress response in ON+CSR group were significantly higher than in ON+PDLI group, which would inevitably lead to longer recovery time of organ function. Another study reported that postoperative mortality was as high as 50% with the aggressive surgical approach [17]. During the ON for LIPN, the colon was edematous grossly and adhered to the surrounding tissue. It is inevitable that the surrounding organs may be damaged during the operation, especially in the hepatic flexure and splenic flexure. In our ON+CSR group, the splenic flexure could not be dissociated from spleen easily in one patient. After separation, hemorrhage occurring in splenic hilum, the spleen and part of the colon were resected together, which increased the operation time and aggravated the surgical trauma. Although the operation was completed, the patient finally died of multiple organ failure after operation. However, in ON+PDLI group, two patients with confirmed colonic necrosis or perforation during operation were performed with CPD via DLIST and PDLI, which not only conformed to the damage control concept, but also made full use of intestinal function after operation. In ON group, 13/23 patients with colonic complication could not made full use of intestinal function after operation for colon in circulation. It revealed that ON patients profited more from the CPD via DLIST and PDLI in terms of duration of organ failure, local and systemic complications. Besides, length of hospital stay was compared in the three groups, despite the additional intraoperative procedures, the ON+PDLI did not prolong and even tended to reduce the length of hospital stay. When ON+PDLI approach administrated, the costs in hospital was markedly reduced, and the time interval from operation to total enteral nutrition was significantly shorten, which implied substantial economic and resource-saving benefits, although there was no significant difference in the mortality.

Patients with LIPN who underwent ON could benefit from CPD + PDLI approach as follows. Continuous positive drainage via a large-bore double-lumen irrigation-suction tube is one of the key techniques to ensure adequate drainage after operation. Early enteral nutrition can be started with PLDI. According to the clinical manifestations, we can adjust the strategy of enteral nutrition timely and safely, which can implement total enteral nutrition as early as possible by keeping colon out of circuit. This is important to protect the gut barrier, reduce bacterial translocation, and even decrease the morbidity and mortality by reducing the septic complications. PDLI can manage the diversion proctocolitis by antegrade succus entericus reinfusion, which may also prevent the colonic complications such as diarrhea, obstruction, missing colon fistula and stenosis after performing closure of the stoma [38, 39]. PDLI could decrease the rate of reoperation, the hospitalization time and cost of hospitalization. It avoids the resection of innocent colon and is more in line with the damage control concept.

There were also some limitations in this study. First, it was a retrospective cohort study with a small sample size. Large samples randomized controlled study should be needed for in-depth investigation in the future. Second, intraoperative bleeding patients were excluded, and the effect of PDLI in these patients has not been evaluated. Furthermore, complications such as post-operative bleeding cannot be avoided, and there is no significant reduction in post-operative mortality. Finally, the time to pulmonary, circulatory and kidney failure were not evaluated in this study. Therefore, whether patients would have developed these outcomes after discharge was still unknown. Further study was needed to evaluate these outcomes.

## Conclusion

Our study suggests that CPD has initially solved the problem of inadequate drainage after ON. Though it may be impossible to control for all the operative and surgical variation that inherently exists in the management of LIPN, our study may provide a new strategy for treatment of LIPN.

## Data Availability

The datasets used or analysed during the current study are available from the corresponding author on reasonable request.

## Abbreviations

LIPN: Late infected pancreatic necrosis
ON: Open necrosectomy
ON+CSR: Open necrosectomy with colonic segment resection
ON+PDLI: Open necrosectomy with prophylactic diverting loop ileostomy
TEN: Total enteral nutrition
CPD: Continuous positive drainage
SAP: Severe acute pancreatitis
DLIST: Double-lumen irrigation–suction tube
DLI: Diverting loop ileostomy
ACS: Abdominal compartment syndrome
BMI: Body mass index
APACHE: Acute physiology and chronic health evaluation
SOFA: Sequential organ failure assessment
CRP: C-reactive protein
PCT: Procalcitonin
WBC: White blood cells
TEN: Total enteral nutrition
CSR: Colonic segmental resection
IQR: Interquartile range
